# Genome-Scale Metabolic Modeling Enables In-Depth Understanding of Big Data

**DOI:** 10.3390/metabo12010014

**Published:** 2021-12-24

**Authors:** Anurag Passi, Juan D. Tibocha-Bonilla, Manish Kumar, Diego Tec-Campos, Karsten Zengler, Cristal Zuniga

**Affiliations:** 1 Department of Pediatrics, University of California, San Diego, 9500 Gilman Drive, La Jolla, CA 92093-0760, USA; anpassi@health.ucsd.edu (A.P.); m3kumar@health.ucsd.edu (M.K.); dcampos@health.ucsd.edu (D.T.-C.); kzengler@eng.ucsd.edu (K.Z.); 2 Bioinformatics and Systems Biology Graduate Program, University of California, San Diego, 9500 Gilman Drive, La Jolla, CA 92093-0760, USA; j1tiboch@eng.ucsd.edu; 3 Facultad de Ingeniería Química, Campus de Ciencias Exactas e Ingenierías, Universidad Autónoma de Yucatán, Merida 97203, Yucatan, Mexico; 4 Department of Bioengineering, University of California, San Diego, La Jolla, CA 92093-0412, USA; 5 Center for Microbiome Innovation, University of California, San Diego, 9500 Gilman Drive, La Jolla, CA 92093-0403, USA

**Keywords:** genome-scale metabolic models, big data, computational tools, phenotypes, flux balance analysis, machine learning, reconstruction, ME-models

## Abstract

Genome-scale metabolic models (GEMs) enable the mathematical simulation of the metabolism of archaea, bacteria, and eukaryotic organisms. GEMs quantitatively define a relationship between genotype and phenotype by contextualizing different types of Big Data (e.g., genomics, metabolomics, and transcriptomics). In this review, we analyze the available Big Data useful for metabolic modeling and compile the available GEM reconstruction tools that integrate Big Data. We also discuss recent applications in industry and research that include predicting phenotypes, elucidating metabolic pathways, producing industry-relevant chemicals, identifying drug targets, and generating knowledge to better understand host-associated diseases. In addition to the up-to-date review of GEMs currently available, we assessed a plethora of tools for developing new GEMs that include macromolecular expression and dynamic resolution. Finally, we provide a perspective in emerging areas, such as annotation, data managing, and machine learning, in which GEMs will play a key role in the further utilization of Big Data.

## 1. Introduction

The beginning of the 21st century has initiated a new era in the generation of Big Data. Major technological advances have enabled the generation of Big Datasets in a cost-efficient and high-throughput manner [1]. Data generated by approaches such as genomics, transcriptomics, proteomics, epigenomics, metabolomics, pharmacogenomics, fluxomics, or phenomics constitutes most of the Big Data in biology and medicine [2]. In simplest terms, Big Data refers to “multi-omics” data that is simply too big and complex for traditional computational tools and resources to be analyzed efficiently [3].

**Figure 1:**
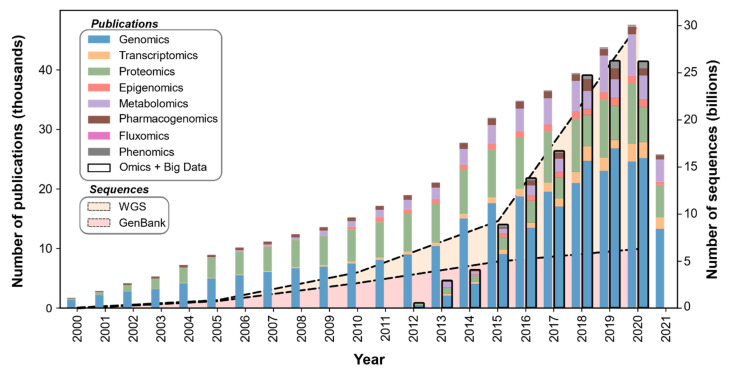
**Rate of publications related to different omics-related fields**. PubMed search results for keywords such as “genomics”, “transcriptomics”, “proteomics”, “epigenomics”, “metabolomics”, “pharmacogenomics”, “fluxomics”, and “phenomics” in publications from 2000–2021. Stacks with “black” borders represent PubMed search results with the keyword “big data” and above-mentioned omics keywords (Supplementary File S1). Moreover, NCBI has added billions of bases to its sequence database over the last decade. It should be noted that the figure does not intend to represent any correlation of publications to the number of sequences.

The initial wave of biological Big Data was powered by the advancement and cost-effectiveness of sequencing technologies, leading to repositories of a large variety of genomes. It served as a foundation for the subsequent waves of omics, which has resulted in a growing wealth of “multi-omics” repositories. The growth of multi-omics Big Data can be perceived through the high number of published multi-omics research. For example, a simple keyword search of different omics research areas on NCBI PubMed [4] such as “genomics”, “transcriptomics”, “proteomics”, “epigenomics”, “metabolomics”, “pharmacogenomics”, “fluxomics”, and “phenomics” reveals an increasing rate of publications over the last two decades in different “omics” research (Figure 1).

**Figure 2:**
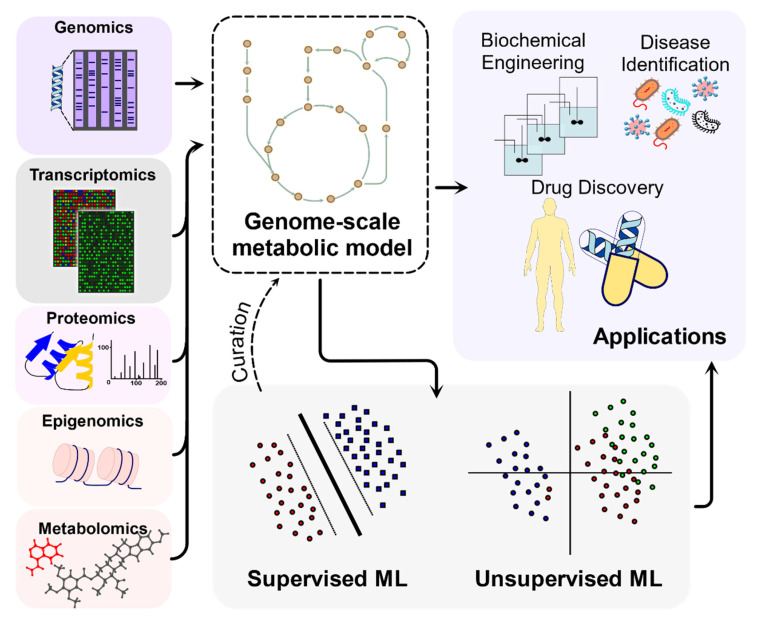
**Big Data types commonly used in metabolic modeling**. The left panel represents different omics data applied to the GEM providing different layers of biological knowledgebase. Machine learning can be applied to increase the predictive capability of the reconstructed GEMs. Different applications of GEMs are shown in the top right panel and discussed in detail in the text.

The exponential increase of Big Data in biology has been challenging to analyze due to the different types of omics data (e.g., by discipline, large variation in data formats, and data structures) [5], lack of metadata (descriptors), and the limited tools to analyze it. Moreover, omics datasets usually require different levels of data scaling, normalization, and transformation [6]. Systems biology and machine learning approaches can help to integrate the different omics datasets to understand the interactions between different cellular components (Figure 2).

Cellular components function through inter- and intra-cellular interactions that can be represented with an “interactome” network in which components such as proteins, genes, metabolites, and other macromolecules are represented as nodes, and the interactions between these cellular components correspond to the edges. These networks can represent transcriptional regulatory networks [7], protein–protein interactions networks [8], disease networks [9], metabolic networks [10], or even host–microbe networks [11]. Genome-Scale Metabolic Models (GEMs) are a network-based tool that collect all known metabolic information of a biological system, including the genes, enzymes, reactions, associated gene-protein-reaction (GPR) rules, and metabolites [12]. These metabolic networks provide quantitative predictions related to growth or cellular fitness based on GPR relationships. GEMs can effectively integrate other types of Big Data to validate metabolic networks that can be used in three broad aspects [13]: (i) understanding the metabolism of archaea, bacteria, fungi, and host organisms like humans and plants [14]; (ii) identifying potential therapeutic targets of disease pathology [15]; and (iii) designing biological systems with preferred features which are otherwise non-existent in nature [16]. They help to understand molecular mechanisms in an organism and identify new processes that might be counter-intuitive to the known biological phenomenon [17,18].

Traditionally, GEMs were developed for individual isolated organisms. However, over the last decades, the study of microbial communities has gained a lot of interest in the scientific community, especially to understand the complex interactions between host organisms and their associated microbiome [19,20,21,22]. GEMs can successfully contextualize microbial omics studies such as metagenenomics, metatranscriptomics, and metabolomics. These complex datasets are now being integrated with other “omics” data to gather insights into the effect of niche microbiota on their hosts [23]. For example, The Human Microbiome Project (HMP) was developed to characterize the human-associated microbiome. Combined, the Human genome and HMP generated 42 terabytes of data [24]. In 2010, the Earth Microbiome Project (EMP) was conceived to systematically characterize the microbiome across the globe [25]. This project has generated over 340 gigabytes of sequencing data, and another 15 terabytes of sequencing and metadata are expected to be generated by the completion of the project [25]. The Vertebrate Genomes Project (VGP) [26], which aims to generate high-quality reference genomes for 70,000 vertebrate species, is expected to generate data in petabytes. Fremin et al. developed MetaRibo-Seq that performs ribosome profiling (Ribo-Seq) of a large number of organisms in a microbiome to measure differences in translation of gene transcripts [27].

Here, we present a comprehensive review of the latest information of biological Big Data and how GEMs are a reliable tool to contextualize and understand them. We discuss how computational tools enable an in-depth understanding of experimental data to accelerate our knowledge of bacteria, archaea, and eukaryotes. We also discuss available tools for reconstructing context-specific GEMs using Big Data [28]. We discuss how biological Big Data has been integrated into GEMs and machine learning tools to enhance their predictive capabilities. Furthermore, we provide a brief overview of the application of GEMs in different areas of research in industry and academia as well as the description of next generation GEMs and future perspectives.

## 2. Individual and Multi-Strain GEMs Connect Genomics with Metabolism

GEMs can be reconstructed using automatic and semi-automated tools. Over 6000 metabolic models have been generated either through semi-automatic or automatic genome-scale reconstruction tools, covering bacteria, archaea, and eukaryotes [29]. GEMs contain all known metabolic reactions and their associated genes of a target organism; the growth rate of the organism is predicted by simulating the metabolic fluxes in the system. Methods available to perform predictions are well-known and include Flux Balance Analysis (FBA), ^13^C-metabolic flux analysis (^13^C MFA), and dynamic FBA (dFBA) [30]. While ^13^C MFA uses labeled isotope tracers to predict the metabolic fluxes, FBA uses measurements of consumption rates as constraints to predict fluxes throughout the entire network [30]. In the coming sections, we discuss various tools that apply FBA to predict the metabolic fluxes under different assumptions. Moreover, we also discuss the concept of dFBA to predict the metabolic fluxes and non-steady-state conditions [31]. Below, we review high-quality models that have been extensively manually curated and validated.

## 3. Multi-Strain Reconstructions of Bacteria Can Help Understand Metabolic Diversity

**Figure 3:**
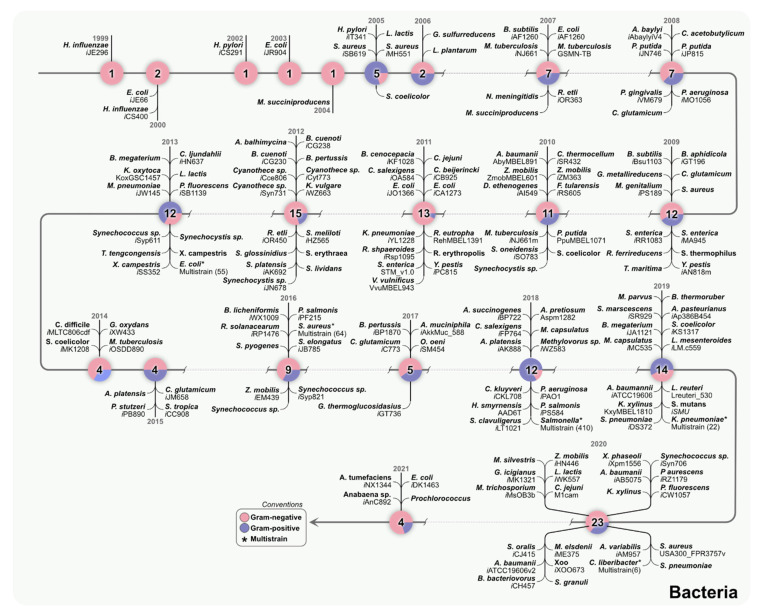
**Reconstructed GEMs for bacteria.** Each node represents a different year. The nodes provide information on the number of reconstructed models and their classification into Gram-negative (pink) and Gram-positive (blue). Some of the organisms like *Escherichia*, *Staphylococcus*, *Klebsiella*, *Liberibacter*, and *Salmonella* also have multi-strain models constructed as represented by asterisk (Supplementary File S2).

Pan-genome analysis unravels variability among genomes of multiple strains, resulting in divergent phenotypes across the strains [32,33]. Based on this concept, GEMs for a single strain can now be expanded to create models for multiple strains of the same species using genomics information [34]. In 2013, Monk et al. created a multi-strain GEM from a set of 55 individual *E*. *coli* GEMs. They created a “core” model that was the intersection of all the genes, reactions, and metabolites of the individual models and a “pan” model that was a union of those models [35]. In another work, Seif et al. developed a *Salmonella* model from 410 individual GEMs of *Salmonella* strains and predicted its growth in 530 different environments [36]. Bosi et al. developed GEMs from 64 strains of *S*. *aureus* and analyzed its growth under 300 different growth conditions [37]. Norsigian et al. reconstructed 22 GEMs of *Klebsiella pneumoniae* strains to simulate growth under 265 different carbon, sulfur, nitrogen, and phosphorus sources [38]. In 2020, Zuniga et al. created a multi-strain GEM from six *Candidatus Liberibacter* asiaticus (*C*Las) strains. They reported conserved and unique metabolic traits, as well as strain-specific interactions between *C*Las and its hosts [14]. These studies advocate in favor of developing multi-strain models for different species that can provide strain-specific insights at network level. Multi-strain GEMs are based on individual GEMs. These expanded modeling analyses lay the foundation for understanding disease-associated traits associated with multi-strain isolates. Figure 3 showcases models that have been reconstructed over the years for different bacteria species, which can serve as primary source of information for multi-strain models. In 2021, Rajput et al. reported the potential of the bacterial two-component system as drug targets by performing a comprehensive pan-genome analysis of ESKAPPE (*Enterococcus faecium*, *Staphylococcus aureus*, *Klebsiella pneumoniae*, *Acinetobacter baumannii*, *Pseudomonas aeruginosa*, *Enterobacter* spp., and *Escherichia coli*) pathogens [39]. Moreover, due to the broad availability of genomics data, it is now possible to identify variations in different strains of the same species hosted by humans or plants.

## 4. Using GEMs to Understand the Metabolism of Archaea

**Figure 4:**
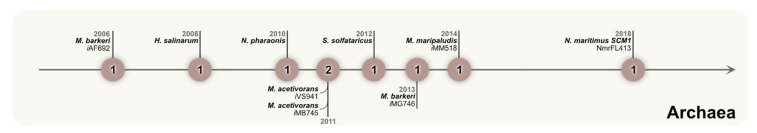
**Available models for Archaea.** The nodes in brown represent the year of GEM reconstruction and number of GEMs reconstructed for archaea (Supplementary File S3).

Archaea are single-cell organisms that contain distinct molecular characteristics from bacteria and eukaryotes. For example, structurally they are associated with bacteria, but evolutionarily they are closer to eukaryotes [40]. As with bacteria, archaea do not contain the peptidoglycan layer in their cell wall but contain a sugar-based polymer [41]. Archaea generate energy differently from other microorganisms and can produce biological methane that bacteria and eukaryotes cannot [42]. Archaea can survive in extreme environments differing in temperatures, acidity, alkalinity, or saltiness. This makes their isolation and studying very difficult. However, archaea are a good source of enzymes that function in extreme temperatures, like Taq polymerases [43]. There are only nine available GEMs of archaea (Figure 4). *Methanobacterium formicicum* (MFI) is a methanogen that is usually present in the digestive system of humans and ruminants [44,45]. It has been implicated in gastrointestinal and metabolic disorders in ruminants, rendering it a clinically important organism. MFI is known to produce methane by utilizing the fermentation products carbon dioxide and hydrogen. There have been five GEMs for members of the family Methanobacteriaceae; namely, *Methanosarcina barkeri* str. Fusaro (*i*AF692 [46], *i*MG746 [47]), *Methanosarcina acetivorans* (*i*MB745 [48], *i*VS941 [49]), and *Methanococcus maripaludis* (*i*MM518 [50]), aiding in our understanding of methanogenesis.

## 5. The Metabolic Complexity of Eukaryotes Is Addressed in GEMs

**Figure 5:**
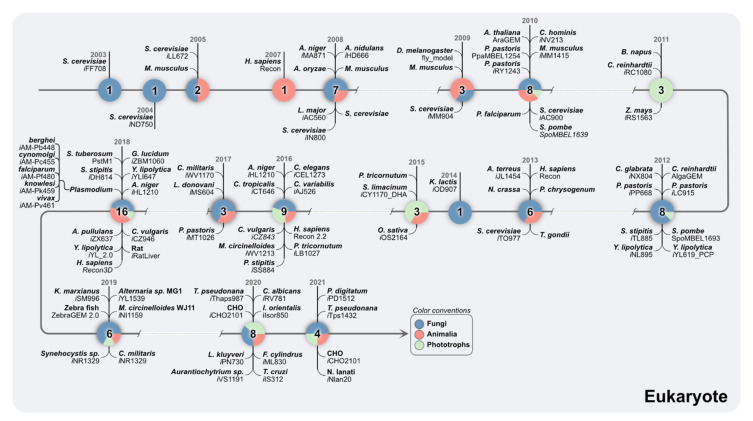
**Chronological order of GEMs of important model eukaryotic** organisms. Each node depicts the year of GEM reconstruction and the number of GEMs reconstructed for that organism. The nodes are color coded to depict the classification of GEMs into Fungi (blue), Animalia (pink) and Phototrophs (green) (Supplementary File S4).

A vast number of modeling efforts have been focused on using novel genomics information of eukaryotic organisms by expanding the number of metabolic networks for a broad range of organisms. Out of the 6000 metabolic models reconstructed to date, a total 215 metabolic models were reconstructions for eukaryotic microorganisms, and only 60 of them have been subjected to manual curation [29]. Figure 5 highlights the eukaryotic organisms with available GEMs. Eukaryotic models are growing both in scale and scope, including organelle-specific metabolic features, multiple compartments, and transport reactions to connect the metabolism across compartments. Expansion of metabolic modeling to eukaryotic organisms envisions their application to increase precursor productiveness for bioenergy [51,52], biocontainment [53], and human health and disease [54,55].

Various computational tools that attempt to predict subcellular localization of proteins have been developed. These include peptide sequence motif prediction (ASAFind) [56], subcellular localization of proteins in different organisms (Cell-PLoc) [57,58], heterokont subcellular targeting (HECTAR) [59], prediction for mitochondrial targeting sequences (MitoProt) [60], prediction of Nuclear Localization Signals (predictNLS) [61], bacterial localization prediction tool (PSORTb) [62], subcellular localization predictor (SCLPred) [63], hybrid subcellular localization predictor (SherLoc2) [64], signal peptide prediction (SignalP) [65], prediction of N-terminal presequences (TargetP) [66], transmembrane helix prediction using hidden Markov model (TMHMM) [67], and protein subcellular localization prediction tool (WoLF PSORT) [68]. Using several of these tools is highly recommended to accurately predict the subcellular localization of proteins from as many compartments as possible. For example, to develop the models of the green algae *Chlorella vulgaris* and *Phaeodactylum tricornutum,* several prediction tools were used (e.g., TargetP, SignalP, HECTAR, Mitoprot, and TMHMM) [69,70].

Six different tools to reconstruct eukaryotic models have been developed so far. For example, (i) AuReMe, which had been tested for eukaryotic algae [71]. The reconstruction process using this tool is based on seven eukaryotic model templates that included two fungi, three algae, one plant and one human model. (ii) The reconstruction capabilities CoReCo [72] were tested by generating 49 fungi models from the divisions Ascomycota, Pezizomycotine, Saccharomycotina, and Basidiomycota using *S*. *cerevisiae* as a template. All models used the same biomass composition of *S*. *cerevisiae* in the modeling reaction. (iii) Merlin [73], which retrieves enzymatic, transport, and localization information from the genome. The program relies on WoLF PSORT to perform this task. Additionally, cross-referencing between Transporter Classification Database (TCDB) [74] and UniProt is performed; however, some ambiguous transporters remain in the reconstructed network. (iv) Pathway Tools is a bioinformatics software that enables reconstruction, prediction of reaction atom mappings, metabolic route search, and regulatory-informatics tools. It contains MetaFlux gap filler that automatically identifies missing reactions, nutrients, and secretions [75]. Finally, (v) the Raven 2.0. toolbox, which performs genome-wide functional annotations, using template models or KEGG as a source for protein homology alignments. The Raven toolbox is currently the most used tool for semi-automatic reconstruction [76]. (vi) The PlantSEED includes genome information of 39 plant and algae species that enable automated annotation and metabolic reconstruction from transcriptome data. PlantSEED reconstructs compartmentalized drafts that can include more than 100 primary metabolic subsystems [77]. The selection of a reconstruction tool for eukaryotic organisms should be an informed decision since reconstruction tools usually have tradeoffs between gapless networks and orphan reactions, meaning that obtaining larger automatic models does not necessarily mean higher quality. Conversely, if the annotation of the genomes is poor, heavy manual curation should be performed. Figure 5 provides a timeline of the eukaryotic GEMs reconstructed to date.

## 6. A growing Branch of Big Data: GEM Reconstruction Tools and Datasets

Emerging applications of GEMs and increased demand for GEMs motivated the generation of automatic and semi-automatic computation tools to generate metabolic models of various organisms from all domains of life. A list of GEM reconstruction tools with their basic properties has been summarized in Table 1. Fundamentally, these tools rely on genome annotations and reaction databases. Genomics data is often available in public domains, such as NCBI Genome [4,78], Ensembl Genome [79], The Encyclopedia of DNA Elements (ENCODE) [80,81], The International Genome Sample Resource (IGSR) [82], or The Database of Genomic Variants (DGV) [83]. In addition to published and curated GEMs, and GEMs available in BiGG [34,84], several reaction databases, such as KEGG REACTION [85], MetaCyc [86,87], MetaNetX [88], Rhea [89], SwissLipids [90], TransportDB [91], and TCDB [74], provide metabolomics and reactions information.

GEM reconstruction tools are distinct from each other due to features like (i) annotation/re-annotation of target genome sequences, (ii) reaction databases, (iii) presence/absence of gap-filling module, (iv) fully-automation or flexibility of customizing parameters, (v) annotation and addition of transport and exchange reactions, (vi) biomass reactions, (vii) presence/absence of subcellular localization module, and (viii) programming language. Additionally, some of them are more used than others, for example, The COBRA toolbox, which has been cited over 2700 times in its three versions (see Table 1).

Most reconstruction tools require an already annotated proteome to map it with reaction databases, whereas tools like merlin and ModelSEED [92] reannotate the genomes before using them in the reconstruction process. Many tools are flexible in terms of using reaction databases; for example, AuReMe [71], GEMsiRV [93], and RAVEN [76] can incorporate the reactions from available GEMs as well as at least one of other reaction databases like KEGG, MetaCyc, BiGG, and ModelSEED. However, the remaining tools only use either available GEMs or other reaction databases; for example, Pathway Tools and ModelSEED only rely on their internal reaction databases. Most of the tools either have a gap-filling module connected with the reconstruction pipeline or as a separate module, except AutoKEGGRec [94], FAME [95], and Pantograph [96], which only provide the draft genome. CarveMe [97], ModelSEED, and Pathway Tools are equipped with an automated pipeline that generates ready-to-use draft models for flux balance analysis. However, more refinement is required to improve the predictive capability of these models and match the quality of manually curated models. The remaining tools allow users to customize any parameters during the reconstruction process or generate a network without biomass, transport, and exchange reactions.

Consequently, merlin encompasses a function to visualize all the reactions in the draft model, and these reactions can also be mapped on the KEGG pathway browser. These functionalities provide opportunities to check and refine reactions and find candidate reactions for filling gaps in the network. RAVEN provides the options to set user-defined template models and blast parameters (i.e., E-value, identity, sequence coverage, and alignment length) during finding the homolog proteins between proteomes of target and template organisms [76]. MetaDraft has in-built manually curated BiGG models in its pipeline, but user-defined template models can also be added in the reconstruction process [98]. GEMsiRV uses user-defined template models, and it extracts the reaction from reaction databases like BiGG, KEGG, MetaCyc, and ModelSEED during the gap-filling process [93].

**Table 1:** Available GEM reconstruction tools and their features.

Tool	Reaction Databases	Advantages/Limitations	Platform	Availability	Citations (Average/Year)	Reference
AuReMe	Available GEMs, MetaCyc, and BiGG	It stores the information at each step during the reconstruction process to maintain transparency and reproducibility.	Docker image	Public	36 (13)	[71]
AutoKEGGRec	KEGG	It can be used to reconstruct models for a single organism and a given list of organisms. It generates an intermediate consolidated model that contains all the genes and reactions for all target organisms. Further, this consolidated model can be used to generate individual models. It does not incorporate transports, exchange, and biomass reactions to the draft model. Gap-filling is also not part of this reconstruction tool.	Matlab	Public	22 (7.33)	[94]
CarveMe	BiGG	It is an automatic tool for reconstructing and gap-filling the draft model. CarveMe generates ready-to-use models for flux balance analysis. As a reaction database, manually curated BiGG models are used in the reconstruction process.	Python	Public	151 (50.33)	[97]
COBRA toolbox, COBRApy, COBRA.ji	-	These tools do not provide any function to build the models based on annotated genomes. However, they provide the functions to incorporate all the components, such as genes, reactions, and metabolites into the model. In particular, these tools are useful for expanding upon existing draft models.	Matlab, Python, and Julia	Public	COBRA toolbox v.1-3.0—2733 (170) COBRApy—612 (76.50) COBRA.ji—25 (6.25)	[99,100,101]
COBRAme	Available GEMs	It is used to develop ME (Metabolism and Expression) models, which are the extended version of GEMs. In addition to a high-quality GEM, these models also contain transcription, translation, and tRNA charging reactions.	Python	Public	73 (24.33)	[102]
CoReCo	Available GEMs, KEGG	It is a comparative reconstruction approach that uses available high-quality GEMs for comparison and reactions from the KEGG database to build models for closely related species. Its capability to compare models makes this tool useful for conducting evolutionary studies.	Python, R, Perl	Public	68 (9.71)	[72]
FAME	KEGG	It only works on the organisms available in the KEGG database. It allows the visualization of FBA results on KEGG pathway maps.	Web-based	Public	93 (10.33)	[95]
GEMsiRV	Available GEMs, BiGG, KEGG, MetaCyc, ModelSEED	It generates the model based on orthologous genes between the target and template model provided by the user. It can perform gap-filling using reference databases from BiGG, KEGG, MetaCyc, and ModelSEED.	Web-based	Public	43 (4.78)	[93]
Merlin	KEGG, TCDB	It comprises several specific features, such as annotation of both enzymatic and transport genes, subcellular localization. Therefore, it can be used to reconstruct the models for both prokaryotes and eukaryotes. This tool also has a function to visualize all reactions in the model that can help users in the gap-filling process using the KEGG pathway browser.	Java	Public	90 (15)	[73]
MetaDraft	Available GEMs	It uses available GEMs as templates to build models for a new organism. It contains internal template models (BiGG models) as reaction databases; however, users can create and use more templates.	Python GUI	Public	28 (7)	[98]
ModelSEED/KBase	ModelSEED	In the first step, it uses RAST to annotate the genome of target organisms. This tool builds the models based on annotated genome and internal reaction databases. It performs gap-filling as a part of an algorithm based on user-provided media or complete media. It is a fully automated tool and does not allow users to customize any steps during reconstruction. It works on the assumption that all the reactions in the internal database are mass and charge-balanced. It also supports model reconstruction for plants.	Web-based	Public	919 (83.55)	[92]
Pantograph	Available GEMs	It uses available models as a reaction database and orthology mappings between genomes of target and template organisms to reconstruct the GEM. It does not apply automatic gap-filling to the draft models.	Python	Public	22 (3.67)	[96]
Pathway Tools	MetaCyc	It generates the model based on genes, reactions, and metabolites stored in organism-specific PGDB (pathway/genome database) and annotated genome. PGDB also helps in filling the gaps in the pathways. It contains 12 experimentally confirmed biomass reactions. Based on the taxonomy of the targeted organism, one biomass reaction is incorporated into the model.	Web-based, Python (via PythonCyc)	Free for academic and government researchers, a license fee applies for commercial use.	216 (43.2)	[75]
RAVEN	Available GEMs, KEGG. MetaCyc	It provides a flexible environment to build a draft model. Users can employ multiple template models simultaneously. This tool can also be used to build the models using reaction databases like KEGG and MetaCyc. Additionally, networks built on different databases can be merged into one model. RAVEN also contains functions for gap-filling and subcellular localization (for eukaryotes).	Matlab	Public	97 (32.33)	[76]
rBioNet	-	This is a part of COBRA Toolbox. It is not an automatic tool to populate the reactions in a draft model from any reaction database. Users need to provide manually or automatically created reaction databases as input for this tool. It comprises the functions to check the quality of newly added reactions such as duplication, charge, and mass balances.	Matlab	Public	71 (7.1)	[103]
SuBliMinal Toolbox	KEGG, MetaCyc	It provides the modules to extract the reactions from KEGG and MetaCyc and merge both versions into a single network. This tool creates biomass reactions based on the biomass precursor present in the draft model. It also has a module to perform subcellular compartmentalization for reactions in the network.	Java	Public	103 (10.3)	[104]

Except for merlin, all reconstruction tools rely on genome annotations, template models, and reaction databases for adding transporter and exchange reactions. Merlin directly annotates the transport genes and reactions using a transporter database, TCDB [74]. For biomass reactions, most tools use the biomass compositions of template models or rarely manually generated reactions based on experimental data of biomass composition. CarveMe uses four template biomass reactions for Gram-positive bacteria, Gram-negative bacteria, Cyanobacteria, or archaea [97,105]. The ModelSEED pipeline uses different biomass reactions for Gram-positive bacteria, Gram-negative bacteria, fungi, and plants. Pathway tools have 12 different biomass reactions based on experimental data from the literature for different taxonomic linkages.

CoReCo contains a function to run comparative analysis on closely related organisms useful for conducting evolutionary studies [72]. FAME can currently only be used to generate models for organisms present in KEGG [95]. One advantage of using this tool is that it can visualize Flux Balance Analysis (FBA) results on KEGG pathway maps, which can help users to interpret flux distribution data. rBioNet is an extension of The COBRA Toolbox [103]. This tool only encompasses the functions for adding model components (genes, reactions, and metabolites) and relies on users to provide data of organism-specific model components. The COBRA toolbox [99], COBRApy [100], and COBRA.ji [101] were mainly developed for reconstructing, reading, editing, and analyzing existing models. However, they have functions to add genes, reactions, and metabolites to the GEMs.

## 7. Integrating Big Data and Machine Learning to Improve Manual Curation of GEMs

As discussed earlier, multi-omics Big Data is expanding at an increasing rate. Machine learning methods have become an essential part to understand and handle the complex nature of Big Data. Recently, machine learning (ML) has been applied to improve the accuracy of GEMs by combining the knowledgebase of the biological system with the predictive power of ML [106]. For example, Ryu et al. developed a deep learning model, DeepEC, using convolutional neural networks (CNN) to predict enzyme commission (EC) numbers and assign those to proteomics information [107]. Schinn et al., developed an integrated machine learning and metabolic model to predict time-course dependent estimation of amino acid concentrations in Chinese Hamster Ovary (CHO) cell cultures [108], an approach validated using metabolomics data.

Unsupervised ML approaches, such as principal component analysis (PCA), and clustering can help in reducing the dimensionality of omics data which can be applied to, for example, identify active reactions in GEMs [109], create subnetworks of genes, and/or metabolic pathways from larger GEMs to answer specific biological questions. Moreover, supervised machine learning approaches such as linear regression, support vector machines (SVM), etc., can infer relationships between different layers of omics data and integrate with GEMs such as identifying essential genes using SVM, and decision trees [110,111], predicting growth and changes in functional states using linear regression [112,113], identifying biochemical effects of antimicrobial resistance causing alleles using hybrid ML and FBA platform [114], etc. Although ML/FBA hybrid models have shown promise in harnessing the biological knowledgebase from omics data and GEMs, there are certain limitations that need to be considered. There is a danger of overfitting of parameters in ML models that reduces the robustness of ML models. Feature selection and cross-validation techniques can be used to avoid overfitting.

The curation of an individual GEM is labor-intensive and time-consuming. The manual curation process can take several months for bacteria and years for eukaryotic organisms. Curation involves adding orphan reactions, refinement of specific model compartments or biomass functions, correct mass imbalanced reactions, etc. [115]. This process highly depends on the intuition of the researcher and standardized methods to select blast parameters and accelerate manual curation. Thus, researchers are developing machine learning algorithms to help prioritize the curation process. These algorithms take advantage of deploying ensemble methods to improve the performance of GEMs. Medlock et al. developed a tool called AMMEDEUS (Automated Metabolic Model Ensemble-Driven Elimination of Uncertainty with Statistical learning) that develops multiple GEMs based on experimental data and simulates these models based on single-gene knockouts. Based on the output, the authors generated similarity profiles based on unsupervised machine learning using cluster analysis. Random Forest classification algorithm was deployed to predict cluster membership based on varying parameters of the model as input. This method helps in identifying parameters that can reduce the uncertainty in the simulation process [115].

In another study, Oyentunde et al. developed a framework called BoostGAPFILL, which uses a combination of constraint-based and pattern-based methods for metabolic model refinement [116]. They used ML to predict a set of possible reactions by characterizing the topology of the incomplete metabolic network. BoostGAPFILL presents 60% precision and recall. Mesquita et al. identified cost-effective ways of measuring low oxygen concentrations, creating a surrogate artificial neural network model by simulations of a GEM. This surrogate model was then used in a fermentation strategy [117]. Culley et al. developed an ML-based method that integrates metabolic models with large-scale gene expression data to understand the different mechanisms of cell growth in 1143 *Saccaromyces cerevisiae* mutant strains [118]. They created 1229 strain-specific models and measured their metabolic activity (fluxomics). They then combined the gene expression and fluxomics data to create predictive models using algorithms, such as support vector regression (SVR), random forest (RF), and artificial neural networks (ANNs), to characterize cell growth [118].

ML techniques have also been used to annotate genes [119]. Stiehler et al. recently developed a platform named Helixer that can improve gene annotations of eukaryotic genomes using deep learning models [120]. Other applications of ML in gene annotation, such as protein-coding gene identification [121], protein function predictions [122], and metabolic pathway prediction [123], have increased the predictive power of GEMs.

## 8. Systems Applications of GEMs Enable a Better Understanding of Big Data

GEMs have become highly relevant during the last decades due to their ability to computationally simulate the complex metabolic processes carried out by different organisms [124]. Metabolic models are currently used to elucidate, comprehend, analyze, optimize, and even discover new cell functions when the studied organisms are subjected to different conditions [124,125]. Some model organisms with high research and industrial value have been updated several times as new genomic, genetic, biochemical, and other biological information became available. For instance, the GEM for *E*. *coli* K-12 MG1655 has constantly been evolving. The initial model contained 660 associated genes [126], while the most recent model more than doubled the genes in the model, containing more than 1500 genes [127].

The continuous updating of GEMs, accompanied by biological Big Data has directly influenced the creation of well-curated modeling databases and tools to integrate the modeling results with omics data. There are databases focused on collecting and retrieving well-constructed and most recent models. The BiGG database compiles high-quality manually curated GEM databases. Additionally, CarveMe, a BIGG-based Database, has emerged as another important modeling database focused on the reconstruction and retrieval of bacteria and archaea microorganisms, facilitating the obtention and simulation of GEMs.

GEMs have varying biological scope and coverage [128]. GEMs might be used for (i) elucidating general metabolic mechanisms of well-studied organisms [129,130,131]; (ii) identify and predict metabolic phenotypes depending on the medium conditions [14,127,132]; (iii) drug discovery and targeting [133,134,135,136]; and (iv) understanding the model interactions between key model organisms and host-microbe interactions [19,137].

## 9. Elucidation of Underground Metabolic Mechanisms of Well-Studied Organisms

Most of the initial GEM reconstructions have been targeted to establish the first models capable of linking the biological data of key organisms with their mathematical and computational representations (in silico). *E*. *coli* K12 MG1655, *Saccharomyces cerevisiae,* and other key organism GEMs have played an important role in understanding general metabolic pathways (glycolysis, pentose phosphate pathway, amino acids metabolism, lipids metabolism, energy core metabolism, etc.) and establishing the important relations among the elements of the GEMs (reactions, genes, metabolites, gene-protein associations, etc.). Based on this mathematical–biological relation, GEMs are used to elucidate the general metabolic mechanisms of the studied organisms using systems biology approaches. For instance, the first GEM of an acetogen, *Clostridium ljungdahlii* DSM 13528 [138], modeled the Wood–Ljungdhal pathway of carbon fixation [138]. GEM of *Azotobacter vinelandii* DJ was developed to elucidate the nitrogen fixation pathway [132]. The predictions of growth rates and internal fluxes based are validated using the available experimental data. The resulting GEMs are usually updated due to the constant renewal of the biological, biochemical, and genomic data available of the key organisms. Most of the GEM updates are focused on bacterial species [97,127] due to the low complexity of the models. However, relevant archaea and eukaryotic organisms are also updated frequently with new GPR associations (gene-protein reactions), reactions, metabolites, genes, or even internal metabolic fluxes.

## 10. Simulation of Phenotypic Traits Depends on the Medium Conditions

GEMs of several organisms have been employed to test the metabolism of a wide range of different nutrients and substrates. Once the metabolic models are built and validated with experimental phenotypic data (growth values, internal fluxes, or expression data), they are usually tested with new carbon, nitrogen, phosphorus, and other elements as substrates to identify the specific mechanisms applied by the organisms to consume these nutrient sources. A recent example is the experimental validation of more than 3000 conditions for *E*. *coli* K12 MG1655 using metabolic modeling predictions [127]. The new updated model (*i*ML1515) is capable of successfully predicting the tested conditions with more than 90% accuracy. Based on the metabolic estimations performed by *i*ML1515, it is possible to establish new biological processes to describe the observed phenotypes. Lu et al. developed a comprehensive *S*. *cerevisiae* metabolic model Yeast8 along with a cluster of metabolic models (ecYeast8, proYeast8DB, panYeast8, and coreYeast8), representing an ecosystem that can be integrated to understand the metabolism of yeast under different carbon and nitrogen sources and understand the genotype–phenotype relationship [139]. Chang et al. developed a GEM for *C*. *reinhardtii* (*i*RC1080) to simulate growth under different light sources. They created photon-utilizing reactions (prism reactions) that represent 11 different light sources used to study plant and algal growth, including solar, LEDs, and other light bulbs [140]. Their platform can help in predicting light source efficiencies related to metabolic objectives.

Another relevant example are GEMs of bacteria with polytrophic metabolism. For example, the well-studied diazotroph bacterium *Azotobacter vinelandii* DJ [132]. More than 40 carbon and nitrogen sources were tested to determine with statistical parameters the quality of the initial predictions. However, the GEM was subsequently validated with over 300 substrates to identify the possible mechanisms employed by this nitrogen-fixing bacterium to consume a wide variety of nutrients. As a result, the model successfully predicted the principal pathways used by *A*. *vinelandii*. The new metabolic processes described to consume the different substrates by the metabolic model agree with the previous experimental data from different approaches (growth, and genomic and fluxomic data). Ultimately, the model operated as a system validator to identify the active metabolic pathways during polyhydroxybutyrate and alginate production (both high-value secondary metabolites) in diazotrophic and non-diazotrophic conditions.

## 11. Utilization of GEMs in Drug Target Identification

GEMs can predict possible biological targets of an organism under a specific condition [141]. The GEM approach has been widely employed to suggest possible metabolic drug targets through inhibition mechanisms to reduce the negative effect or kill the pathogen. Developing a comprehensive metabolic network can also help identify potential novel drug targets that can kill disease-causing pathogens. Recently, Viana et al. constructed a GEM of the human pathogen *Candida albicans* (*i*RV781) with 1221 reactions, 781 genes, and 926 metabolites [142]. They identified 11 ERG genes that guide the ergosterol biosynthesis in the organism, and targeting the ERG pathway mimicked the effects of a fungicide. In 2019, Minato et al. used *Mycobacterium tuberculosis* GEM *i*SM810 to predict essential genes that can be potential drug targets [143]. In another work, Wang et al. developed a GEM for the plant pathogen *Pectobacterium carotovorum* (*i*PC1209) that contains 2235 reactions, 1113 metabolites, and 1209 genes [144]. They identified 19 potential bactericide targets among essential genes through simulating single gene deletions in the metabolic model. Haleem et al. developed a highly complex GEM of *Plasmodium falciparum* (*i*AM-Pf480), representing five life cycles of the malaria-causing pathogen [145]. They report 95% accuracy in predicting single-gene knockouts and 71% accuracy in predicting drug inhibition phenotypes. They identify 48 genes that can be potential drug targets for malaria [145]. Weglarz-Tomczak et al. developed a novel method called Gene Expression and Nutrients Simultaneous Integration (GENSI) for the human reconstruction Recon3D that uses gene expression data and nutrient availability data and converts it into fluxes. The study explored the effect of diet on cancer cell metabolism and the rate of progression [146]. In another study, Puniya et al. developed a GEM to identify possible drug targets for CD4^+^ T cell-mediated diseases. They first identified essential genes and then perturbed the network using existing Food and Drug Administration (FDA) approved drugs and compounds. They were able to identify 55 potential drug targets for three autoimmune diseases, such as rheumatoid arthritis (RA), multiple sclerosis (MS), and primary biliary cholangitis (PBC) [147]. These studies highlight the potential of GEMs to become an integral part in identifying novel therapeutic targets. However, experimental validation of these drug targets can be a challenging task.

## 12. Contextualization of Disease-Associated Big Data—Systems Medicine

A disease phenotype is usually a result of perturbations in cellular interaction networks, not only due to an abnormal gene [148]. Systems approaches help understand these cellular networks and a particular disease and provide potential drug targets. GEMs have an equally useful role in understanding human metabolism and, in turn, human diseases. There have been many research studies that employ GEMs to understand various cancers. Nilsson et al. presented a comprehensive review on methods applied to generate GEMs in cancer research [149]. Pandey et al. analyzed different subtypes of renal cell carcinoma using the transcriptomics data in conjunction with human GEM. They identified alterations related to amino acid metabolism, redox homeostasis, glycolysis, and TCA cycle in cancer subtypes [150]. Gatto et al. assessed how cancer-specific GEMs differ from normal tissue GEMs. They were able to identify reactions catalyzed by ARG2, RHAG, SLC6 and SLC16 family gene members, and prostaglandin-endoperoxide synthase (PTGS1 and PTGS2) were exclusively present in cancer models. However, their findings suggest a vast similarity between cancer-specific GEMs and normal tissue GEMs, and targeting tumor metabolism could cause toxicity as the GEMs have the same underlying metabolic functions [151].

GEMs have been deployed to identify biomarkers for complex diseases such as cancers. In cancer, there are genetic and epigenetic alterations in the metabolism. By incorporating omics data into the metabolic models, cancer biomarkers can be predicted by estimating the exchange rates of different metabolites in the model [152]. To understand changes in brain metabolism under disease conditions, Moolmalla et al. reconstructed GEMs for three psychiatric disorders: schizophrenia, bipolar disorder, and major depressive disorder, and compared it with the human Recon3D model [153]. By applying transcriptomics data to the models, they were able to identify alterations between the three psychiatric disorders at flux level [153].

## 13. Multi-Level Integration of Big Data in Emergent Modeling Approaches

The acceleration of GEM reconstruction across several biological domains gave rise to new questions that could previously not be answered by GEMs, such as dynamic functional states and macromolecular expression. For example, dynamic metabolic models have been successfully used to characterize growth dynamics, time-dependent cycles, and organelle crosstalk [21]. On the other hand, the integration of additional biological layers to GEMs allowed addressing macromolecular expression. This section reviews the resulting hybrid models developed to address these questions, their implications, and principal findings.

## 14. Adding Macromolecular Resolution—Proteometrics

GEM-PRO models contain detailed annotation of protein structure without altering either the metabolic network or the numerical strategy to find metabolic flux distributions through Flux Balance Analysis (FBA). In a GEM-PRO model, structure annotations are added as a new layer on top of the biochemical reaction network, which allows for a systems-level analysis of protein structure trends within the network and the predicted metabolic fluxes. The first GEM-PRO model was generated for *Thermotoga maritima*, which included protein sequence and fold annotations [154]. These annotations helped address the mechanism of pathway evolution by discovering that enzymes catalyzing similar reactions have a significantly higher probability of exhibiting the same fold. This finding reported that new biochemical reactions are likely attained by recruiting an enzyme from an existing similar reaction.

The following GEM-PRO models were generated for *Escherichia coli* [155,156]. The first included a protein-ligand interaction network with resolution of binding sites at residue level. This study coupled protein structure with protein–ligand predictions using the SMAP method to identify antibacterial targets and complexes with potential antibacterial properties. In another study, transcriptomics at 37 °C and 42 °C were analyzed for heat-induced gene expression. The expression of these genes, deemed part of the heat-shock response, were used to constrain the *E*. *coli* GEM at different temperatures [156]. This model was employed to predict mutations and metabolite supplements that would induce thermotolerance in *E*. *coli* identifying growth-limiting proteins and their associated pathways.

A similar, but more detailed, GEM-PRO with a residue-level resolution of protein structure was generated for a human GEM in Recon3D [133]. The model further includes three-dimensional data on residue spatial position in the protein, which was successfully employed to identify mutation sites that induce conformational changes. Interestingly, Recon3D successfully captured those mutations within 10 Å of the metal-binding site of arylsulfatase A induce its homo-dimer state, which directly alters the stability of this protein and is linked with a mild form of metachromatic leukodystrophy [157]. GEM-PRO models have not only been used to improve the analysis of metabolic networks and fluxes, but also to guide model reconstruction. For example, protein structures were used to identify enzyme homologs for the GEM-PRO of *Staphylococcus aureus* [158].

## 15. Simulating Gene Expression of Cells

Another approach to include macromolecular information to GEMs was realized with the introduction of models of metabolism and gene expression (ME-models). In this case, the metabolic network itself is altered by adding reactions for enzyme synthesis and assembly proportional to the flux of the catalyzed metabolic reaction. The coupling of metabolic reactions with protein synthesis allows the calculation of a systems-level protein synthesis profile, which directly informs about the proteome composition of the organism with a particular metabolic phenotype [102]. Moreover, this coupling adds a biosynthetic requirement to the metabolic fluxes, reducing the variability of fluxes [159] and eliminating unbound fluxes with previously no biological relevance. The Toolbox COBRAme for python was developed to create ME-models. COBRAme does not have functions to create a GEM from scratch, however the code can be adjusted to different organisms.

The coefficients of proportionality between coupled reactions are called coupling coefficients derived from enzyme kinetics of catalysis and degradation, and their dilution to newly produced biomass. The first ME-model was reconstructed for *T*. *maritima* [159], which defined the necessary coupling constraints for complex usage, transcription, translation, and mRNA degradation. This model successfully reproduced amino acid consumption, peptide translation, and transcription rates under different growth conditions.

The following ME-models were reconstructed for *E*. *coli* in four iterations, namely Thiele et al. [160], *i*OL1650-ME [128], *i*JL1678-ME [161], and *i*JL1678b-ME [102]. The model by Thiele et al. [160] correctly captured experimental growth rates in different carbon sources, their codon usage and increased the accuracy of gene essentiality predictions. Next, *i*OL1650-ME successfully captured RNA-protein ratios at varying growth rates, as well as glucose uptake rates and phosphotransferase enzymatic activities. The effect of nitrogen, sulfur, phosphorus, and magnesium levels on growth rate was correctly captured by *i*OL1650-ME. Further, the model identified three growth modes resulting from nutrient availability: nutrient-limited, proteome-limited, and a transition between both. Third, *i*JL1678-ME [161] accounted for protein translocation pathways, which allowed it to predict proteome allocation in different compartments and the inner membrane occupation in response to metabolic phenotypes.

The main limitation of these ME-models was their solution complexity and stability due to them being nonlinear large optimization problems, with over 70,000 reactions. The solveME package [162] was generated to increase accuracy and improve scaling in the system by using the binary search algorithm and quad-precision in the calculations. The most recent *E*. *coli* ME-model, *i*JL1678b-ME [102], drastically reduced the number of reactions by reformulation the coupling coefficients, from 79,871 [128] and 70,751 [161] reactions in previous iterations to just 12,655 reactions. The reformulation consisted mainly of combining subreactions into a single reaction and effectively deriving new coupling coefficients for each resulting reactant and product. *i*JL1678b-ME proved to be as accurate in its translation and transcription rate predictions as its predecessors and more so in the gene essentiality predictions, in only a fraction of the solution time. The COBRAme toolbox was used to reconstruct the ME-model of *Clostridium ljungdahlii* [163], which predicted transcription rates highly correlated with experimental transcriptomics. Moreover, this model accurately simulated the effect of trace metal concentrations, such as nickel, on the growth rate.

Lately, additional biological and biochemical layers have been added to ME-models to simulate the effect of stress conditions, e.g., temperature, pH, and oxidative stress. FoldME [164] integrated folding and degradation kinetics to predict the effect of temperature on growth rate, effectively predicting low- and high-pH stress, as well as the optimal pH range. AcidifyME [165] coupled folding and unfolding thermodynamics and kinetics and was able to predict variation in lipid composition (characterized by a notable increase in cyclopropane), periplasmic protein stability, and membrane protein activity. Finally, OxidizeME [166] integrated kinetics of iron–sulfur cluster damage and repair, as well as metalation and mismetalation, to predict differential expression under high levels of reactive oxygen species.

## 16. Overcoming the Steady-State Assumption in Genome-Scale Metabolic Models

The steady-state assumption of FBA limits GEMs to capturing growth at a particular time during culture, though critical biochemical phenomena may occur in a time-dependent manner. Dynamic Flux Balance Analysis (dFBA) was the first approach to address non-steady-state simulations using FBA and GEMs. Mahadevan et al. [31] first proposed two formulations for dFBA: static and dynamic optimization approaches (SOA and DOA). The SOA consists of a forward numerical method with a defined time-step, where uptake rates are calculated using the steady-state assumption at each step and concentrations are updated. The SOA was later expanded by Zhao et al. [167] using a nonlinear objective function.

On the other hand, the DOA alters the definition of the optimization function, where the new objective function is a concentration integrated over a timespan, e.g., the total production of biomass. Further work on the DOA was performed by Zhou et al. [168] by using an exterior penalty function to improve the accuracy of predictions. Thus, while the DOA is more robust, the SOA is much less computationally intensive.

A third strategy to solve dFBA was proposed by Höffner et al. [169] and is available in the MATLAB package DFBAlab [170], where lexicographic optimization (called the Direct Approach or DA) is employed instead of the traditional SOA and DOA. DA solves the previously existing issue of flux non-uniqueness by sequentially optimizing the objective function and the exchange rates. DFAlab was shown to capture growth dynamics in batch fermentation with *Saccharomyces cerevisiae* and *E*. *coli* [170].

Even though dFBA can obtain stable and unique solutions of time-course concentrations, especially during nutrient-replete conditions, it alone cannot capture sub-optimal growth under stress or nutrient limitation. The underlying optimization problem of dFBA exchange fluxes is constrained by either observed fluxes in vivo or unconstrained. Naturally, flux uptake and secretion rate limitations must vary with time during the culture timespan. This led to the hybrid dFBA systems constrained by kinetic models of uptake and secretion, called multiscale models [171,172]. A dFBA approach was employed by Kuriya et al. [173], where models with fitted parameters constrained glucose and biomass concentrations.

Multiscale models have been generated for the photosynthetic microalga *Chlorella vulgaris* coupled with kinetic models to predict growth dynamics. Chien-Ting et al. [51] constrained the growth rate of *C*. *vulgaris* GEM *i*CZ946 [113] with time-course growth rate data. This model was employed to optimize nutrient supply to maximize growth and lipid productivity.

Nonetheless, multiscale models are not limited to the simulation of sub-optimal growth, as any other model can be coupled with a GEM to capture the desired phenomenon [172]. A multiscale model of yeast, GECKO [174], integrated enzyme kinetic models to calculate enzyme abundances from metabolic fluxes, which were then constrained by experimental values. A similar approach was employed by Chen et al. [175] to calculate enzyme-binding metal ions and assess metabolic responses to ion limitation.

GEMs can also be given dynamicity using the biomass objective function (BOF), especially in organisms with drastically changing biomass compositions, such as photosynthetic microalgae [56]. In a study by Zuniga et al. [113], the biomass composition of *C*. *vulgaris* was measured during batch culture and was integrated into the GEM *i*CZ946. The resulting model accurately predicted growth rate under nitrogen-replete and nitrogen-deplete conditions and discovered a nitrogen pool in the microalga. A similar strategy was employed by Tibocha-Bonilla et al. [52] on five different eukaryotes (including two microalgae and two yeasts) to predict time-course organelle and pathway activities. In another study, time-course *chlorophyll a* absorption coefficients and abundances were used to constrain the GEM of the diatom *Phaeodactylum tricornutum*, thus capturing circadian clock oscillations and discovering mechanisms to release excess reducing power [176]. Moreover, van Tol et al. [177] measured biomass compositions of *Thalassiosira pseudonana* under three light levels and integrated them in its GEM, effectively predicting the effect of light intensity on the growth rate. Furthermore, the model predicted the contributions of the cyclic and non-cyclic electron flows to the total electron flow.

## 17. Challenges Associated with Reconstruction of GEM and Omics Data Integration

Network-based tools have shown to be a reliable tool for big data analysis and contextualization. Different methods for metabolic flux analysis such as FBA, 13C MFA, and dFBA have some limitations. First, FBA assumes that the system is under steady-state [178]. Second, the FBA solution can contain loops limiting accuracy in predicting anaplerotic, circular, and parallel reactions [179]. FBA cannot predict metabolite concentrations as it does not employ kinetic parameters [30]. Moreover, it does not account for the regulation of gene expression [30]. Also, FBA has to deal with the inherent issues of alternate optimal solutions, where a different set of fluxes of reactions in the metabolic network can be used to get the same quantitative values of the objective function (e.g., cell growth) [180]. One way of identifying the alternate optimal solutions is to identify the variability of fluxes to understand the boundaries of entire solution space instead of relying on one solution and then assessing which of those solutions are favorable for the model system. Flux Variability Analysis (FVA) [180], Flux Coupling Analysis (FCA) [181], and Comprehensive Polyhedron Enumeration FBA (CoPE-FBA) [182] are some of the approaches that can be utilized for this purpose. Another approach can be to utilize random sampling to calculate different flux distributions under varying constraints and experimental conditions [183].

Most of the constraint-based modeling methods can be seamlessly applied to prokaryotes. However, eukaryotic models and other non-model organisms are quite complex to reconstruct due to the lack of complete genome assemblies, diverse secondary metabolites and their intracellular complexity organized by compartments/organelles, such as cytoplasm, chloroplast, mitochondria, nucleus, periplasm, peroxisome, and thylakoid [184]. Recent efforts in improving the sub-cellular localization of proteins have been continuously enhancing the quality of metabolic contents in GEMs of eukaryotes [185]. The simulation capabilities of automatically generated GEMs are usually limited. This may primarily be due to a lack of good quality sequence and annotation data. Draft GEMs can have inaccurate information of biomass reactions and GPR associations [29]. The quality of GEMs can be enhanced using semi-automatic approaches that combine manual curation and experimental evidence [132]. Moreover, for a high-quality GEM, it is imperative that draft GEM reconstruction follow the Findability, Accessibility, Interoperability, and Reuse (FAIR) guiding principles for scientific data management [186,187]. The network entities (genes, metabolites, and reactions) should be *findable* using unique identifiers and mapped to known databases. The model should be *accessible* for the users to make and retrieve significant changes to draft reconstruction. The draft GEMs should be written in standard SBML formats. Moreover, the steps involved at different stages of draft reconstruction should be transparent to users so that the GEM is reusable and reproducible [188].

Parameter estimation and model fitting is another major challenge in effectively utilizing GEMs. Constraint based GEMs which are based on linear programming do not include any time dimensions and do not account of metabolite concentrations [189]. Dynamic/kinetic constraint-based GEMs apply enzyme kinetics to increase the scope of these models. However, dynamic models include large numbers of enzyme kinetic parameters that usually cannot be estimated directly. Moreover, depending upon the size of the models, parameterization of kinetic models can be time consuming and computationally expensive. Due to this, kinetic models are still not as accepted as constraint based GEMs [190]. However, there are some efforts to make kinetic models more acceptable to the modeling community by creating sub-networks of a bigger model where kinetic parameters can be fitted easily [189].

Another major challenge is the integration of omics data. As omics datasets represent different aspects of biological systems, there are challenges in developing a credible knowledge base for integration in GEMs, such as non-uniform and missing data, inefficient computation power to analyze omics data, signal-to-noise ratio in the data, inconsistent annotations, or storage and distribution of data [191]. Moreover, it is difficult to integrate omics data from different studies due to the variation in sample handling, sequencing depth, and limited availability of metadata information [192]. Preprocessing of data, including data normalization, bias removal, and quality checks can help overcome these limitations [192]. Further, noise is diminished by using omics data from studies that use similar omics technologies, materials, established standard operating procedures, and references [193]. As GEM reconstruction usually depends on homology prediction, it can fail in identifying characteristic metabolic features of organisms that are phylogenetically or functionally different from the well-characterized model organisms [194].

Despite the challenges associated with omics data integration with GEMs, each integrated layer of omics data helps in minimizing metabolic gaps and providing realistic predictions for organism specific cellular metabolism [194].

It is well known that a model cannot predict and mirror the observations from experimental observations with 100% accuracy. Many models try to be more biologically relevant by adding experimental data based on various growth environments. Moreover, the models are updated as and when the biological information is available that provides new insights into the metabolism of a particular organism. For example, iML1515 is the most updated model of *E*. *coli* [127] with 2719 reactions and 1192 metabolites. Since *E*. *coli* is a model organism, new biological information is constantly being reported that helps in updating the model with a higher frequency. iML1515 contains 184 new genes and 196 new reactions compared to the older version. This is now the benchmark model for E.coli and, perhaps, can predict with higher accuracy comparable to experimental observations [127]. This effort can also guide other organism’s models towards improving the accuracy of their predictive capabilities.

## 18. Conclusions and Perspectives

Big Data has enabled the fast development of systems biology tools. These advancements have triggered the reconstruction of genome-scale metabolic models for a wide range of organisms and applications. In this review, we have presented the current state of metabolic modeling in the context of biological Big Data. We have provided a comprehensive account of existing GEMs that utilize the vast repertoire of multi-omics data, available tools to reconstruct those GEMs, and their applications in different fields of biological research. GEMs are proven and robust platforms to understand the complex metabolic processes of biological organisms. Although, there are certain challenges associated with storing, analyzing, and interpreting Big Data to create a valuable knowledgebase, computational algorithms for data compression, distributed storage databases, and cloud computing can aid in solving these challenges [195]. As the data grows, the complexity, scope, and scale of the GEMs will continue expanding. Traditionally, the GEMs have all been assumed to be working under steady-state conditions. New studies are now providing a dynamic state to the GEMs to understand the metabolic pathways in a time-dependent manner. GEMs have found their applications in essentially every aspect of biological research including elucidating core metabolic pathways, gene essentiality, functional annotations, industrial applications, drug discovery, and host-microbe interactions.

Since integration of biological Big Data provides several layers of biological knowledgebase to the GEMs, such as protein, the applications of GEMs can go beyond just understanding metabolic systems to include other host systems like the nervous systems or the immune system. This will help in understanding diseases linked to microbial environments, impact of probiotics [196], and diet modulation on diseases like autism [197], obesity [198], etc. Keeping in mind the technical challenges associated with Big Data and GEM reconstruction, there is considerable evidence that GEMs will be applied in understanding an expanding range of complex interactions between different biological systems.

## Supplementary Materials

The following supporting information can be downloaded at: https://www.mdpi.com/article/10.3390/metabo12010014/s1.

## Abbreviations

GEM	Genome-Scale Metabolic Model
GSMMs	Genome-scale metabolic models
NCBI	National Centre for Biotechnology Information
ENCODE	Encyclopedia of DNA Elements
DGV	Database of Genomic Variants
KEGG	Kyoto Encyclopedia of Genes and Genomes
RAVEN	Reconstruction, Analysis and Visualization of Metabolic Networks
COBRA	Constraint-Based Reconstruction and Analysis Toolbox
TCDB	Transporter Classification Database
FBA	Flux Balance Analysis
MFI	*Methanobacterium formicicum*
